# Normalization of the Mini-MAC (Mental Adjustment to Cancer) Questionnaire among Cancer Patients

**DOI:** 10.3390/ijerph182312603

**Published:** 2021-11-29

**Authors:** Aleksandra Czerw, Urszula Religioni, Filip Szymański, Agnieszka Nieradko-Heluszko, Dominika Mękal, Dagmara Hering, Anna Kowalczuk, Piotr Merks, Mariola Borowska, Magdalena Bogdan, Monika Pajewska

**Affiliations:** 1 Department of Health Economics and Medical Law, Medical University of Warsaw, 02-091 Warsaw, Poland; mariola.borowska@wum.edu.pl; 2 Department of Economic and System Analyses, National Institute of Public Health—NIH—National Research Institute, 00-791 Warsaw, Poland; mpajewska@pzh.gov.pl; 3 Collegium of Business Administration, Warsaw School of Economics, 02-513 Warsaw, Poland; urszula.religioni@gmail.com; 4 Departament of Civilization Diseases, Faculty of Medicine, Collegium Medicum, Cardinal Stefan Wyszyński University in Warsaw, Kazimierza Woycickiego 1 Street, 01-938 Warsaw, Poland; f.szymanski@uksw.edu.pl; 5 Subdepartment of Social Medicine and Public Health, Department of Social Medicine, Pomeranian Medical University in Szczecin, 70-103 Szczecin, Poland; agnieszka.nieradko.heluszko@pum.edu.pl; 6 Department of Oncological Prevention, Medical University of Warsaw, 02-091 Warsaw, Poland; dmekal@wum.edu.pl; 7 Department of Hypertension and Diabetology, Faculty of Medicine, Medical University of Gdansk, 80-211 Gdansk, Poland; hering@gumed.edu.pl; 8 National Institute of Medicines, 00-725 Warsaw, Poland; a.kowalczuk@nil.gov.pl; 9 Department of Pharmacology and Clinical Pharmacology, Faculty of Medicine, Collegium Medicum, Cardinal Stefan Wyszyński University in Warsaw, 01-938 Warszawa, Poland; piotrmerks@googlemail.com; 10 Department of Social Medicine and Public Health, Medical University of Warsaw, 02-007 Warsaw, Poland; mminko@onet.eu

**Keywords:** normalization, mental adjustment, cancer, mini-MAC

## Abstract

Cancer is associated with discomfort and many changes in patients’ lives to which they must adapt. The main objective of the study was to assess the use of the mini-MAC questionnaire scale among persons diagnosed with malignant cancer and to develop standards allowing differentiation of patients with diagnosed cancer in terms of their style of adjustment to the disease. The mini-MAC questionnaire is a widely used tool in assessing coping strategies among cancer patients. Sten standards have been developed to determine the level of results on the questionnaire scales in the low–average–high categories. The study included 1187 patients diagnosed with malignant cancer who are covered by outpatient care at the Maria Sklodowska-Curie Institute—Oncology Center in Warsaw. The questionnaire concerning mental adjustment to cancer was used (mini-MAC). Patients with cancer most often adopt strategies of fighting spirit and positive reevaluation. The variables that differentiate the results most significantly include gender, presence of metastasis, and the state of undergoing chemotherapy. The mini-MAC questionnaire should be a tool for psycho-oncological diagnosis of patients’ attitudes towards cancer. The obtained results indicate that cancer patients are characterized by their constructive style of adjustment to the disease.

## 1. Introduction

Diagnosis and treatment of cancer is usually associated with many negative experiences for the patient [1]. Chronic diseases result in the occurrence of chronic stress for the patient, as well as changes in lifestyle and social roles, and experiencing pain and discomfort, to which the patient must adapt. Mental adjustment to the disease is a process aimed at removing emotional discomfort and restoring a state of mental balance of a person suffering from cancer. Adjustment to the disease is used to cope directly with the disease, but also with situations related to the disease, e.g., treatment or changes in the patient’s life [2].

The model of adjusting to cancer developed by Watson et al. includes five main adjustment attitudes: fighting spirit, avoidance–denial, fatalism–stoic acceptance, helplessness–hopelessness, anxiety [3]. Research conducted using this model indicates that fighting spirit is associated with low external control and high social support, while helplessness–hopelessness—with high external control and low social support. Helplessness–hopelessness manifests itself with a sense of hopelessness and helplessness, passivity, anxiety, low spirits, and depression in patients, while anxiety manifests itself with an anxious attitude towards the diagnosis and the entire therapeutic process, as well as, among others, with hypochondrial behavior [2,3,4]. In turn, patients using constructive strategies to deal with cancer are characterized by a higher quality of life and a better prognosis regarding survival and remission periods [2,5,6].

The mini-Mental Adjustment to Cancer (mini-MAC) questionnaire is a well-recognized and used tool in measuring coping strategies among cancer patients in five basic areas [7]. The mini-MAC questionnaire has been translated and adapted in many countries, including in China [8], Portugal [9], Italy [10], Greece [11], Korea [12], Iran [13], and Norway [14].

Although the original version of the mini-MAC test covered five areas of mental adjustment to the disease, validation studies justify four areas [15]. Studies using the mini-MAC questionnaire are usually conducted on small groups of patients [16]; therefore, it is necessary to check repeatability of results obtained on a large group of patients.

The main objective of the study was to assess the appropriateness of using the mini-MAC questionnaire, measuring the level of adjustment to the disease among patients with malignant cancers.

## 2. Materials and Methods

### 2.1. Characteristics of the Studied Normalization Group

The study is prospective in nature. The criteria for enrolling patients were the patient’s availability at the Maria Sklodowska-Curie Institute—Oncology Center in Warsaw during conducting the study and the patient’s consent to participate in the study. The study involved 1187 people diagnosed with cancer, including 56.1% women and 43.9% men. The mini-MAC questionnaire is based on 29 items which gave us the ratio of more than 40 participants for one item. The need to acquire a large sample was motivated by the goal of calculating normalized scores. Among women, the most common cancers in the study group were breast cancer, ovarian cancer, endometrial cancer, and colorectal cancer, while among men: prostate cancer, colorectal cancer, bladder cancer, and stomach cancer (Table 1).

The studied patients were 21–96 years old (M = 62.12; SD = 14.03) and they had mainly secondary or higher education. Most people lived in cities with >500,000 residents. Most people were pensioners/retirees, but professionally active people also constituted a large group. Most patients were married (Table 2).

### 2.2. Applied Tool

The mini-MAC questionnaire, developed in Polish by Z. Juczyński, is based on the Mental Adjustment to Cancer Scale [17], constructed by Watson et al. The mini-MAC questionnaire consists of 29 statements, measuring four ways of dealing with cancer [18]:

1. Anxiety—related to fear concerning the disease that is seen as a threat that cannot be controlled and therefore causes anxiety.
2. Fighting spirit—making the patient treat the disease as a challenge and take action to fight it.
3. Helplessness–hopelessness—creating a sense of powerlessness and passive submission to the disease.
4. Positive reevaluation—causing the patient during his/her sickness to feel satisfied with the years lived so far.

While fighting spirit and positive reevaluation constitute part of the constructive style of coping with the disease, anxiety and helplessness–hopelessness are components of the destructive style.

Each statement is rated on a scale of 1 (definitely not) to 4 (definitely yes), and the results available for each of the four strategies for dealing with the disease are 7–28 points. The higher the score, the greater the severity of the behaviors in the patient’s struggle with cancer.

Cronbach’s alpha coefficients for individual strategies range from 0.87 (for positive reevaluation) to 0.92 (for helplessness–hopelessness).

In the study, statistical analyses were performed to verify the impact of socioeconomic variables on the obtained test results in patients. Statistical analysis was performed with the use of IBM SPSS 25.0 software (IBM, Armonk, NY, USA). The analysis regarding between-groups comparisons was based on the values of independent t test when comparing two groups (gender, city/town size, net income, working people vs. retirees/pensioners, patients with/without metastases, patients undergoing vs. not undergoing chemotherapy, radiotherapy, and targeted therapy) and on the values of one-way analysis of variance when analyzing education. When analyzing variables without normal distribution (fighting spirit, helplessness–hopelessness and positive reevaluation), nonparametric tests were used instead. Mann–Whitney U-test was used instead of independent *t*-test, and Kruskal–Wallis H test was used instead of analysis of variance. The groups of participants with primary and vocational education were combined in order to avoid analyzing very small groups (participant’s primary education). Students, homemakers, and unemployed patients were excluded from the analysis concerning professional status due to small number of participants in these three groups.

## 3. Results

Strategies of fighting spirit and positive reevaluation dominate in cancer patients. Table 3, Table 4, Table 5 and Table 6 present descriptive statistics for each of the mini-MAC areas depending on demographic and medical variables. The results indicate that the average value of anxiety depends on gender (*p* = 0.006), size of the place of residence (*p* = 0.021), and income (*p* = 0.035). Strategies of fighting spirit and helplessness–hopelessness were differentiated by the patient’s gender (*p* = 0.001 for fighting spirit and *p* = 0.003 for helplessness–hopelessness). Positive reevaluation depended on gender (*p* = 0.001), education (*p* = 0.018), and professional status (*p* = 0.001).

In terms of the impact of the presence of metastases or the use of specific treatment on mental adjustment to the disease, it was noted that anxiety is differentiated by metastases (*p* = 0.008) and undergoing chemotherapeutic treatment (*p* = 0.003). Fighting spirit is differentiated by metastasis (*p* = 0.017), and helplessness–hopelessness depends on the occurrence of metastases (*p* = 0.01), chemotherapeutic treatment (*p* = 0.03), and targeted treatment (*p* = 0.029). Positive reevaluation is not differentiated by any of the medical variables studied.

### Norms

Due to statistically significant differences between the genders in terms of the results on all analyzed mini-MAC scales, the norms of the questionnaire results were developed separately for women and men. Table 7 and Table 8 show the values of sten and centile norms determined in groups of women and men using the calculated weight for the results of each of the areas of the mini-MAC questionnaire. Sten scores from 1–3 should be interpreted as low, from 4–7 as average, and from 8–10 as high.

## 4. Discussion

Changes caused by cancer result not only in physical and social difficulties for the patient, but also in psychological disorders resulting from chronic stress, which most often include depression, anxiety, and sleep disorders. It is indicated that depression occurs in 20–40% of cancer patients, and sleep disorders in up to 50% of patients [19].

Research indicates that active strategies increase the quality of life, and high levels of helplessness–hopelessness are associated with low quality of life. In addition, the level of social functioning decreases as the intensity of anxiety increases, and in the case of growth in the intensity of helplessness–hopelessness, the level of professional, cognitive, and social functioning decreases [20,21]. In turn, patients intensely applying constructive strategies of coping with the disease function better physically and socially compared with patients showing low and medium intensity of constructive strategy of coping with the disease, while patients showing medium and high intensity of constructive strategies of coping with the disease demonstrate better emotional and cognitive functioning [2].

In the study of mental adjustment of cancer patients, Kulpa et al. indicated that positive reevaluation at a high level was present in 74% of people, and its average result was M = 20.7. Fighting spirit at a high level was present in 66% of patients, and the average result for this strategy was M = 19.75, which is a high score [2]. Anxiety at a high level was present in 47% of respondents, and the average result for anxiety was M = 18.6, which is a moderate result. A high level of helplessness–hopelessness occurred in 31% of patients, and the average result for this strategy was M = 15.9, which is an average result. The overall average intensity of the constructive strategies and of the destructive strategies was the same for both strategies M = 40.4 [2]. The authors’ study indicates a slightly higher result achieved for the constructive strategies and a lower result for the destructive strategies.

Active strategies dominate among people with prostate cancer, and the study conducted by Kulpa et al. indicates that the destructive strategies are associated with a higher severity of anxiety and a greater propensity to perceive a situation as threatening [6].

Dominating strategies applied to adjust to the disease among patients with gynecological cancers include fighting spirit (M = 21.51; SD = 2.96) and positive reevaluation (M = 21.45; SD = 2.46). The median value of anxiety was M = 17.36 (SD = 4.40), and of helplessness–hopelessness—M = 13.87 (SD = 4.04). The results of this study indicate that respondents whose illness lasted more than two years more often (*p* = 0.003) used the strategy of helplessness–hopelessness than those who had been ill for less than two years, and women who did not experience complications during treatment showed a stronger fighting spirit than those who experienced complications (*p* = 0.05) [22].

Similarly, in the case of women with cervical cancer after surgical treatment, it was indicated that constructive strategies prevailed: fighting spirit (M = 22.63; SD = 2.88) and positive reevaluation (M = 21.10; SD = 2.64), while the destructive strategies reached the values of: anxiety M = 16.07 (SD = 4.42) and helplessness–hopelessness M = 12.63 (SD = 3.76) [23].

However, Rogala et al., studying women with breast cancer, did not indicate differences in the mental adjustment to the disease depending on the length of the disease or other factors studied (age, marital status, place of residence) [23]. The studied women were primarily characterized by fighting spirit (M = 23.9; SD = 2.8) and positive reevaluation (M = 23.5; SD = 2.8). Anxiety reached an average of M = 15.6 (SD = 4.6), and helplessness–hopelessness of M = 12.0 (SD = 3.6) [24].

Sobieralska-Michalak et al., assessing mental adjustment among breast cancer patients depending on the type of surgery (amputation or conservative surgery), indicated that both groups of patients choose the constructive strategies to adjust to cancer [25]. Similar results for particular strategies were obtained by Szczepańska-Gieracha et al., comparing mental adjustment to the disease of patients with breast cancer and cancer of reproductive organs, where there were also no significant differences between the studied groups [26].

In patients with colorectal cancer treated with chemotherapy, the results were also similar to those obtained by other authors: fighting spirit M = 23.9, positive reevaluation M = 22.5, anxiety M = 16.1, and helplessness–hopelessness M = 12.3 [27].

The authors’ research results show that in cancer patients, strategies of fighting spirit and positive reevaluation are dominant. The study conducted by Krawczyk et al. showed that patients with lung cancer are also characterized by fighting spirit (M = 23.00; SD = 3.27) and positive reevaluation (M = 21.69; SD = 2.39), while strategies of anxiety and helplessness–hopelessness achieved much lower values (respectively, M = 15.94; SD = 3.72 for anxiety and M = 12.39; SD = 3.32 for helplessness–hopelessness) [28].

Fighting spirit also dominates in patients with head and neck cancers (M = 25.0; SD = 2.6). Patients with head and neck cancers choose positive reevaluation (M = 23.6, SD = 2.2), followed by anxiety (M = 16.1; SD = 4.8) and helplessness–hopelessness (M = 12.5; SD = 3.3) as a strategy to adjust to the disease [29].

In turn, patients with laryngeal cancer in the study conducted by Humeniuk et al. achieved the highest value of the mini-MAC questionnaire in the areas of anxiety (M = 21.8), and positive reevaluation (M = 21.4). Better adjustment to the disease was observed among women, people with higher education, and living in stable relationships with children [30].

In the study of patients with various types of cancer, the average results of individual adjustment strategies were as follows: fighting spirit M = 22.91 (SD = 3.24), positive reevaluation M = 22.07 (SD = 2.67), anxiety M = 19.25 (SD = 3.08), helplessness–hopelessness M = 16.72 (SD = 3.38), and the choice of strategy was not influenced by the studied sociodemographic factors [31].

However, in the study conducted by Baczewska et al., it was found that in the case of adjusting to cancer by patients undergoing chemotherapy, the choice of strategy is differentiated by gender and age. In this study, women demonstrated significantly more intense helplessness–hopelessness and anxiety (*p* = 0.034) and a sense of helplessness–hopelessness (*p* = 0.017) increased with age, with a simultaneous decrease in the value of fighting spirit (*p* = 0.022) [32]. Intensity of the destructive strategies, anxiety, and depression in women is also indicated in other studies, e.g., Ziętalewicz et al. [33].

In the study conducted among people with newly diagnosed thyroid cancer and its recurrence, it was proven that in the group of people with newly diagnosed disease, the destructive strategy was low (M = 31.62), and the constructive strategy was assessed as high (M = 44.6). In patients experiencing recurrence, the destructive strategy prevailed over the constructive one, with M = 45.26 and M = 39.88, respectively [34].

Studying patients with diagnosed stomach cancer, cancer of reproductive organs, pancreatic cancer, colorectal cancer, and prostate cancer under palliative care in hospital and at home, Kozak emphasizes the intensification of helplessness–hopelessness, which was the dominant strategy for this group (M = 19.89; SD = 6.74) [35]. It was observed that men with prostate cancer showed the highest intensity of anxiety, while women with cancer of reproductive organs—the lowest; fighting spirit was a dominating strategy among them. The strategy of helplessness–hopelessness also occurred at the highest intensity in patients with prostate cancer, and in the lowest—in women with gynecological cancers [35].

Studies of young cancer patients indicate that the most frequently chosen strategy for the adjustment to the disease was fighting spirit (M = 23.2); the constructive style was dominant, and gender, place of residence, or duration of the disease did not differentiate the results obtained [36].

Kapela et al. indicated that the manner of mental adjustment to the disease is affected by the degree of its acceptance—the higher the level of acceptance of the disease, the higher the level on the scale of fighting spirit and the higher the level on the scale of the constructive style [27]. Milaniak et al. indicated the influence of optimism on the chosen strategies of mental adjustment to the disease, in particular fighting spirit [29].

Numerous authors proved the relationship between the chosen style of adjustment to cancer and the quality of life, indicating that patients using the constructive strategies assessed their quality of life as higher, and passive attitudes, including anxiety or helplessness–hopelessness negatively affects the patients’ quality of life [2,3,4,37,38]. Anxiety and helplessness–hopelessness additionally exacerbate the symptoms of depression and fear in patients [39,40].

The effect of attitude towards the disease on patients’ prognosis in terms of survival and remission periods was also proven. Fighting spirit results in higher remission and survival rates than the destructive and passive strategies [3,6,17]. Fighting spirit also affects the better functioning of patients in the physical, emotional, cognitive, and social areas compared to those choosing the strategy of helplessness–hopelessness in the face of illness [2]. However, Boryczko-Pater et al. indicated that the style of adjustment to cancer can change over time towards more active strategies [41].

## 5. Conclusions

This study, involving a large part of the Polish cancer patient population, was aimed at establishing the standards of the mini-MAC questionnaire. This information will allow to identify methods of mental adaptation to cancer by patients, and thus to support them in the greatest and most optimal way by clinical psychologists. The mini-MAC questionnaire should be a tool for psycho-oncological diagnosis of patients’ attitudes towards cancer. The obtained results indicate that cancer patients are characterized by the constructive style of adjustment to the disease. However, there are individual differences between them. The developed standards allow for the determination of the level of adjustment to cancer in clinical practice. 

The manner of adjusting to cancer not only affects the patients’ quality of life but is also associated with the results of treatment. The assessment of the style of adjustment to the disease, as well as the intensity of this style, constitutes a valuable hint for medical staff in the decision to include active psychotherapeutic support in the field of work on negative attitudes towards the disease in the treatment process. The patient’s cooperation with a psychologist, aimed at changing the style of adjustment to the disease towards a constructive one, will also improve the overall clinical results.

## Figures and Tables

**Table 1:** Types of cancer in the sample and in the population.

	Women			Men		
Cancer Type	Population (%)	Sample (%)	Weight	Population (%)	Sample (%)	Weight
breast cancer	21.9	29.0	0.76	0	0	-
ovarian cancer	4.7	25.8	0.18	0	0	-
stomach cancer	2.4	6.8	0.35	4.5	9.2	0.49
colorectal cancer	10.1	14.9	0.68	12.2	26.9	0.45
prostate cancer	0	0	-	15.5	43.8	0.35
bladder cancer	2.0	2.6	0.77	6.9	15.9	0.43
endometrial cancer	7.3	17.4	0.42	0	0	-
pancreatic cancer	2.2	3.6	0.61	2.3	4.2	0.55

**Table 2:** Characteristics of the studied normalization group.

Characteristic	*n*	%
Education		
Primary	97	8.3
Vocational	250	21.1
Secondary	447	37.7
Higher	393	33.1
Place of residence		
Village	221	18.6
City up to 20,000 residents	131	11.0
City up to 50,000 residents	154	13.0
City up to 100,000 residents	144	12.1
City up to 500,000 residents	110	9.3
City above 500,000 residents	427	36.0
Average monthly income		
No data	8	0.7
Below PLN 500	24	2.0
PLN 501–1000	210	17.7
PLN 1001–1500	317	26.7
PLN 1501–2000	299	25.2
Above PLN 2000	329	27.7
Professional status		
Working	464	39.1
Student	19	1.6
Pensioner/retiree	618	52.1
Homemaker	53	4.5
Unemployed	33	2.8
Marital status		
Single	93	7.8
Married	812	68.4
Widow/widower	192	16.2
Divorced	90	7.6
Total	1187	100

**Table 3:** Descriptive statistics for the level of anxiety depending on demographic and medical variables.

		M	SD	min	max	Test	*p*
Gender	Women	16.47	4.84	7	28	*t*(550) = 2.77	0.006
	Men	15.31	4.77	7	28		
Education	Prim./voc.	16.49	4.61	7	28	*F*(2.549) = 1.63	0.197
	Secondary	16.06	5.01	7	28		
	Higher	15.54	4.82	7	28		
Size of	Up to 100,000 residents	16.45	4.76	7	28	*t*(550) = 2.32	0.021
city	Above 100,000 residents	15.49	4.90	7	28		
Net	Up to PLN 1500	16.46	4.76	7	28	*t*(550) = 2.12	0.035
income	Above PLN 1500	15.59	4.89	7	28		
Professional	Working	16.06	4.57	7	28	*t*(485.81) = 0.52	0.601
status	Student	15.38	3.80	7	24		
	Pensioner/retiree	15.83	5.05	7	28		
	Homemaker	17.04	4.96	8	25		
	Unemployed	17.46	5.07	7	26		
	Total	16.01	4.84	7	28		
Metastases	yes	16.75	4.87	7	28	*t*(516) = 2.68	0.008
	no	15.54	4.73	7	28		
Chemotherapy	yes	16.82	4.78	7	28	*t*(550) = 2.98	0.003
	no	15.55	4.82	7	28		
Radiotherapy	yes	16.41	4.78	7	27	*t*(550) = 0.74	0.457
	no	15.95	4.86	7	28		
Targeted	yes	17.12	4.20	7	25	*t*(549) = 1.52	0.129
therapy	no	15.92	4.89	7	28		

M—median value; SD—standard deviation; min—minimum value; max—maximum value; *t*—Student *t*-test value for independent samples; *F*—one-way analysis of variance value; *p*—statistical significance.

**Table 4:** Descriptive statistics for the level of fighting spirit depending on demographic variables.

		M	SD	min	max	Test	*p*
Gender	Women	23.01	3.66	7	28	*U* = 1518.00	0.001
	Men	22.14	3.68	12	28		
Education	Prim./voc.	22.79	3.77	11	28	χ^2^(2) = 2.43	0.296
	Secondary	22.71	3.82	9	28		
	higher	22.51	3.47	7	28		
Size of	Up to 100,000 residents	22.72	3.66	8	28	*U* = 15,260.00	0.737
city	Above 100,000 residents	22.61	3.73	7	28		
Net	Up to PLN 1500	22.69	3.71	8	28	*U* = 14,613.50	0.269
income	Above PLN 1500	22.65	3.68	7	28		
Professional	Working	22.59	3.44	7	28	*U* = 11,651.50	0.141
status	Student	22.48	3.31	18	27		
	Pensioner/retiree	22.77	3.85	8	28		
	Homemaker	22.44	3.97	15	28		
	Unemployed	22.42	4.19	10	28		
	Total	22.67	3.69	7	28		
Metastases	Yes	22.42	3.94	8	28	*U* = 10,541.00	0.017
	No	22.86	3.54	9	28		
Chemotherapy	Yes	22.44	3.96	7	28	*U* = 13,357.50	0.216
	No	22.80	3.53	9	28		
Radiotherapy	Yes	22.51	4.43	9	28	*U* = 7751.00	0.852
	No	22.69	3.57	7	28		
Targeted	Yes	22.10	3.46	15	28	*U* = 3902.50	0.193
therapy	No	22.71	3.71	7	28		

M—median value; SD—standard deviation; min—minimum value; max—maximum value; *U*—Mann–Whitney U-test value; χ^2^—Kruskal–Wallis H-test value; *p*—statistical significance.

**Table 5:** Descriptive statistics for the severity of helplessness–hopelessness depending on demographic and medical variables.

		M	SD	min	max	Test	*p*
Gender	Women	12.51	4.34	7	28	*U* = 2267.00	0.003
	Men	12.61	4.39	7	28		
Education	Prim./voc.	13.18	4.61	7	28	χ^2^(2) = 2.08	0.354
	Secondary	12.52	4.20	7	27		
	Higher	12.03	4.26	7	28		
Size of	Up to 100,000 residents	12.68	4.44	7	28	*U* = 14,927.50	0.495
city	Above 100,000 residents	12.38	4.25	7	28		
Net	Up to PLN 1500	13.08	4.43	7	28	*U* = 13,970.50	0.076
income	Above PLN 1500	12.05	4.23	7	28		
Professional	Working	12.24	4.14	7	28	*U* = 12,010.50	0.299
status	Student	13.69	3.31	9	20		
	Pensioner/retiree	12.69	4.43	7	28		
	Homemaker	13.01	5.33	7	28		
	Unemployed	12.95	4.71	7	27		
	Total	12.55	4.35	7	28		
Metastases	Yes	13.42	4.68	7	28	*U* = 9164.50	0.001
	No	12.07	4.11	7	28		
Chemotherapy	Yes	13.32	4.56	7	28	*U* = 11,701.00	0.003
	No	12.10	4.17	7	28		
Radiotherapy	Yes	13.46	4.43	7	25	*U* = 7040.50	0.219
	No	12.41	4.33	7	28		
Targeted	Yes	14.13	4.21	7	24	*U* = 3445.50	0.029
therapy	No	12.43	4.34	7	28		

M—median value; SD—standard deviation; min—minimum value; max—maximum value; *U*—Mann–Whitney U-test value; χ^2^—Kruskal–Wallis H-test value; *p*—statistical significance.

**Table 6:** Descriptive statistics for the level of positive reevaluation depending on demographic and medical variables.

		M	SD	min	max	Test	*p*
Gender	Women	21.99	3.03	7	28	*U* = 1559.00	0.001
	Men	21.45	3.19	10	28		
Education	Prim./voc.	22.20	2.98	11	28	χ^2^(2) = 8.05	0.018
	Secondary	21.93	2.95	12	28		
	Higher	21.22	3.31	7	28		
Size of	Up to 100,000 residents	21.93	3.03	11	28	*U* = 14,938.50	0.501
city	Above 100,000 residents	21.59	3.17	7	28		
Net	Up to PLN 1500	22.06	2.92	11	28	*U* = 13,839.00	0.055
income	Above PLN 1500	21.51	3.24	7	28		
Professional	Working	21.11	3.18	7	28	*U* = 9750.50	0.001
status	Student	21.75	2.90	17	27		
	Pensioner/retiree	22.28	2.98	10	28		
	Homemaker	21.51	2.91	16	27		
	Unemployed	22.29	3.05	16	28		
	Total	21.78	3.10	7	28		
Metastases	Yes	21.78	3.16	10	28	*U* = 12,267.50	0.728
	No	21.84	3.04	11	28		
Chemotherapy	Yes	21.72	3.20	7	28	*U* = 13,826.00	0.479
	No	21.81	3.04	11	28		
Radiotherapy	Yes	21.60	3.55	12	28	*U* = 7431.00	0.511
	No	21.80	3.03	7	28		
Targeted	Yes	21.51	3.21	12	28	*U* = 4258.00	0.537
therapy	No	21.80	3.10	7	28		

M—median value; SD—standard deviation; min—minimum value; max—maximum value; *U*—Mann–Whitney U-test value; χ^2^—Kruskal–Wallis H-test value; *p*—statistical significance.

**Table 7:** Raw results and corresponding results normalized for the results of the mini-MAC questionnaire in the group of women.

Anxiety				Fighting Spirit				Helplessness–Hopelessness				Positive Revaluation			
Results	Value	Sten	Centile	Results	Value	Sten	Centile	Results	Value	Sten	Centile	Results	Value	Sten	Centile
low	7	1	2	low	7	1	1	low	7	2	6	low	7	1	1
	8	2	5		8	1	1	average	8	4	17		10	1	1
	9	3	8		9	1	1		9	4	25		13	1	1
	10	3	13		10	1	1		10	5	33		14	1	1
average	11	4	17		11	1	1		11	5	42		15	1	2
	12	4	20		12	1	1		12	6	51		16	2	4
	13	4	24		13	1	1		13	6	59		17	2	5
	14	4	29		14	1	2		14	6	68		18	3	10
	15	5	37		15	2	3		15	7	75	average	19	4	17
	16	5	44		16	2	5		16	7	80		20	4	27
	17	6	51		17	2	7	high	17	8	85		21	5	37
	18	6	60		18	3	10		18	8	88		22	5	48
	19	6	68		19	3	15		19	8	91		23	6	60
	20	7	76	average	20	4	19		20	9	93		24	7	70
	21	7	83		21	4	25		21	9	95		25	7	83
high	22	8	88		22	5	33		22	9	97	high	26	8	93
	23	8	91		23	5	44		23	10	98		27	9	97
	24	9	93		24	6	57		24	10	99		28	11	99
	25	9	96		25	6	68		25	10	99		25	10	99
	26	10	98		26	7	78		26	10	100		26	10	100
	27	10	99	high	27	8	85		27	10	100		27	10	100
	28	10	100		28	9	95		28	10	100		28	10	100

**Table 8:** Raw results and corresponding results normalized for the results of the mini-MAC questionnaire in the group of men.

Anxiety				Fighting Spirit				Helplessness–Hopelessness				Positive Revaluation			
Results	Value	Sten	Centile	Results	Value	Sten	Centile	Results	Value	Sten	Centile	Results	Value	Sten	Centile
low	7	2	3	low	12	1	1	low	7	3	7	low	10	1	1
	8	3	7		13	1	1	average	8	4	18		11	1	1
	9	3	11		14	1	1		9	4	24		12	1	1
	10	3	16		15	2	3		10	5	32		13	1	1
average	11	4	21		16	2	6		11	5	41		14	2	2
	12	4	27		17	3	9		12	6	51		15	2	3
	13	5	34		18	3	14		13	6	58		16	2	5
	14	5	40	average	19	4	22		14	6	66		17	3	8
	15	5	48		20	4	30		15	7	73		18	3	13
	16	6	56		21	5	38		16	7	77	average	19	4	21
	17	6	63		22	5	47		17	7	81		20	5	32
	18	7	70		23	6	56	high	18	8	86		21	5	43
	19	7	76		24	6	66		19	8	90		22	6	54
	20	7	82		25	7	75		20	9	94		23	6	65
high	21	8	87		26	7	82		21	9	96		24	7	75
	22	8	92	high	27	8	89		22	9	97	high	25	8	86
	23	9	95		28	9	96		23	10	98		26	9	94
	24	9	97						25	10	99		27	10	98
	25	10	98						26	10	99		28	10	99
	27	10	99						27	10	100				
	28	10	99						28	10	100				
